# Le nitrate double NaRb_2_(NO_3_)_3_, composé intermédiaire du système binaire isobare NaNO_3_ + RbNO_3_: études thermiques et cristallographiques

**DOI:** 10.1107/S2056989015006532

**Published:** 2015-04-09

**Authors:** Nesrine Ksiksi, Mohamed Driss, Dalila Hellali, Abderrahmen Guesmi, Hmida Zamali

**Affiliations:** a Université de Tunis El Manar, Faculté des Sciences, Laboratoire de Thermodynamique appliquée, El Manar II, 2092 Tunis, Tunisie; b Université de Tunis El Manar, Faculté des Sciences, Laboratoire de Matériaux et Cristallochimie, El Manar II, 2092 Tunis, Tunisie; c Université de Tunis El Manar, Institut Préparatoire aux Etudes d’Ingénieurs d’El Manar, El Manar II, 2092 Tunis, Tunisie

**Keywords:** crystal structure, NaNO_3_ + RbNO_3_ phase diagram, bond-valence sum, charge-distribution method

## Abstract

Crystallographic and thermodynamic investigations of the binary (NaNO_3_ + RbNO_3_) phase diagram at atmospheric pressure reveal the existence of an inter­mediate compound NaRb_2_(NO_3_)_3_ (sodium dirubidium trinitrate) previously predicted and now reported experimentally for the first time. According to a DSC analysis, the compound exhibits three allotropic forms. In its low-temperature allotropic form (α form, ortho­rhom­bic) there are two Rb (*m*.. site symmetry) and one Na (*m*..) independent crystallographic positions and three planar nitrate groups. The bond-valence-sum calculations for all atoms agree well with their oxidation states. The Rb cations are located in the (100) plane at *x* = ½ with 11 oxygen coordination. The Na ones are in the same plane at *x* = 0 and are coordinated to eight O atoms from six nitrate groups. The charge-distribution method has been used to evaluate the degree of distortion of the alkali polyhedra.

## Abstract 2

NaRb_2_(NO_3_)_3_ nitrate is the only inter­mediate compound in the system NaNO_3_ + RbNO_3_. It has, according to a thermal study, three allotropic forms. The structure of the low-temperature form is presented.

## Contexte chimique   

Les nitrates alcalins sont connus pour leurs propriétés phys­iques importantes. En effet, ils sont utilisés comme fondants à cause de leurs basses températures de fusion, mais ils sont utilisés également dans des électrolytes solides grâce à leurs importantes conductivités ioniques à températue ambiante (Rao *et al.* 2005 ▸). L’association de deux cations alcalins dans un même matériau peut induire de nouvelles propriétés, ce qui nécessite une étude préalable du diagramme de phases des nitrates correspondants. Dans ce contexte nous avons exploré le système NaNO_3_ + RbNO_3_ où on a relevé l’existence du composé inter­mediaire étudié. Par ailleurs le système binaire NaNO_3_ + RbNO_3_ a fait l’objet de plusieurs travaux antérieurs (Diogenov & Sarapulova, 1965 ▸; Cingolani *et al.* 1972 ▸; Sangster, 2000 ▸). Le désaccord entre les différents travaux était au niveau de la formule du composé intermédiaire défini: Diogenov & Sarapulova (1965 ▸) et Cingolani *et al.* (1972 ▸) ont proposé la formule NaRb_2_(NO_3_)_3_ alors que Sangster (2000 ▸) a proposé une stoechiométrie plus riche en rubidium, soit la formule NaRb_3_(NO_3_)_4_. Nos récents résultats obtenus par des études thermiques et de diffraction des rayons X confirment la formule exacte de ce nitrate: NaRb_2_(NO_3_)_3_.

## Analyse thermique   

**Figure 1:**
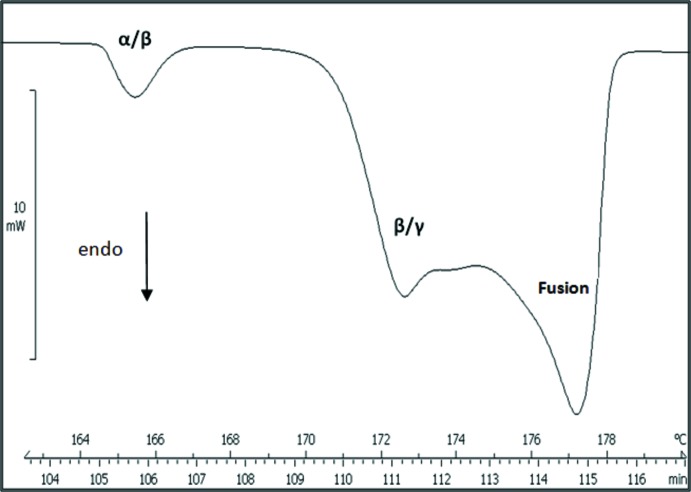
Courbe DSC pour NaRb_2_(NO_3_)_3_ en montée de température (2 K min^−1^).

Les deux nitrates limites possèdent chacun plusieurs formes allotropiques, deux pour le nitrate de sodium et cinq pour le nitrate de rubidium. Afin de mettre en évidence l’existence d’éventuelles transitions de phase pour le composé étudié, nous avons réalisé une étude DSC en montée de température (Fig. 1 ▸). Nous avons déduit de ces mesures que le nitrate double étudié possède trois formes allotropiques α, β et γ qui n’ont pas été signalées auparavant. Les températures de changement de phase sont les suivantes: *T*_Tr._(α→β) = 436 (1) K; *T*_Tr._(β→γ) = 442 (1) K et *T*_Fus._ = 451 (1) K.

## Commentaire structural   

**Figure 2:**
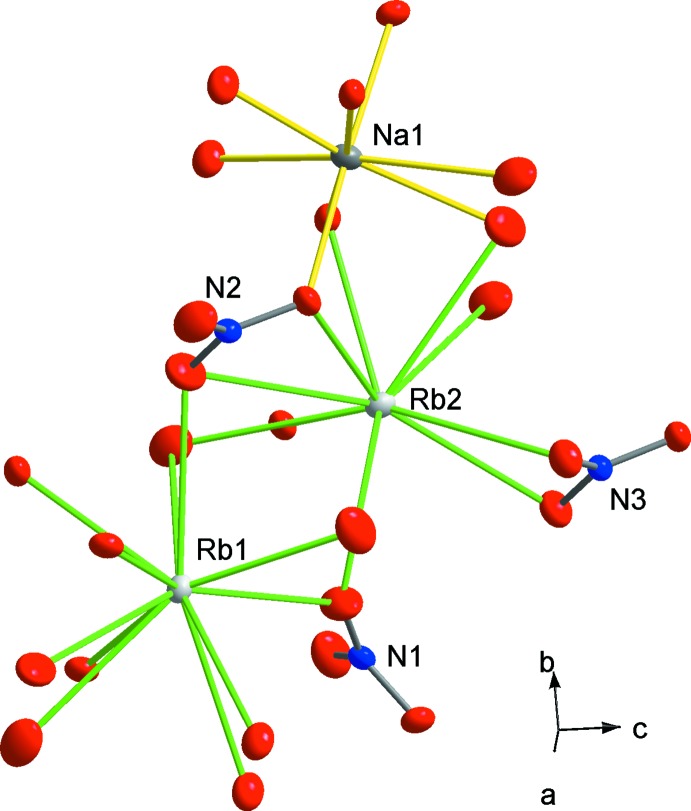
Modes de jonction entre les groupements nitrate et les polyèdres de coordination des cations alcalins (ellipsoïdes d’agitation thermique à 30% de probabilité de présence).

La forme basse température (forme α) cristallise dans le groupe d’espace *Pmc*2_1_. Par ailleurs, l’une des formes allotropiques du composé limite au rubidium cristallise dans le groupe *Pmmn* (Kalliomäki & Meisalo, 1979 ▸). L’unité asymétrique du nitrate étudié renferme un cation Na^+^, deux cations Rb^+^ et trois groupements nitrate de géometrie plane (Fig. 2 ▸). Les valences des cations ainsi que celles des atomes d’oxygène sont en bon accord avec leurs degrés d’oxydation (Adams, 2004 ▸).

**Figure 3:**
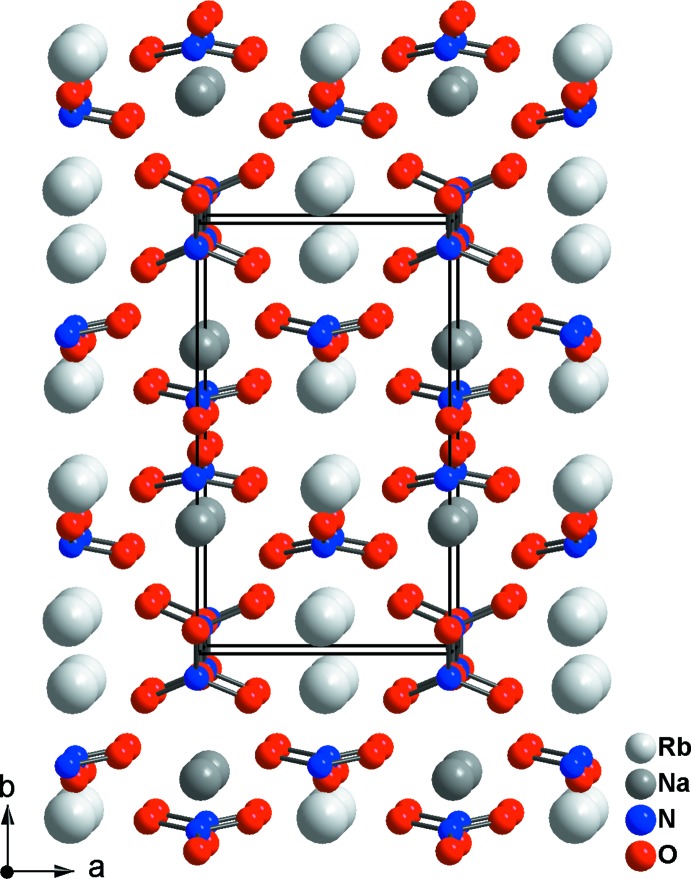
La structure du nitrate étudié vue selon une direction proche de [001].

Les deux cations alcalins occupent des plans perpendiculaires à [100], à *x* = 0 pour Na^+^ avec une coordinence de 8 et à *x* = ½ pour Rb^+^ avec une coordinence de 11 atomes d’oxygène (Fig. 3 ▸). La grande différence entre les rayons ioniques des deux cations explique l’absence d’un désordre de substitution.

**Figure 4:**
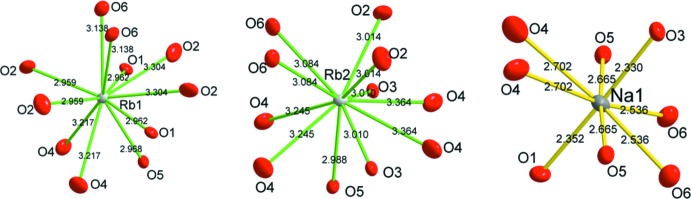
Les polyèdres de coordination des cations alcalins avec leurs distances correspondantes (les ellipsoïdes d’agitation thermique sont à 30% de probabilité de présence).

Les polyèdres de coordination des cations alcalins sont assez distordus, comme c’est généralement le cas pour ce genre de cations. La Fig. 4 ▸ montre ces polyèdres avec les distances correspondantes, les écarts entre la distance la plus longue et la plus courte pour chaque polyèdre sont 0.34 Å pour Rb1, 0.38 Å pour Rb2 et 0.37 Å pour Na1. Pour évaluer leur distortion, nous avons examiné ces polyèdres par la méthode de distribution de charge *CHARDI-IT* (Nespolo, 2001 ▸; Nespolo *et al.* 2001 ▸)*.* Cette méthode a montré d’une part comme la méthode BVS des ‘charges’ en bon accord avec les degrés d’oxydation de tous les atomes et d’autre part des nombres de coordination effectifs (ECoN; Hoppe, 1979 ▸) qui évaluent les degrés de distortion de ces polyèdres: plus l′ECoN s’écarte du nombre de coordination classique CN plus la distortion est importante; les valeurs obtenues sont les suivantes: ECoN/CN(Rb1) = 10.24/11; ECoN/CN(Rb2) = 10.27/11 et ECoN/CN(Na1) = 7.03/8. Les ECoNs des groupements nitrate correspondent bien à la valeur idéale (CN = 3).

## Synthèse et cristallisation   

Le composé étudié a été préparé à partir d’un mélange stoechimétrique des deux nitrates alcalins correspondants. Après fusion, le mélange réactionnel a subi plusieurs cyles successifs de chauffage–refroidissement entre 298 et 473 K, température légèrement supérieure à celle de fusion du composé intermédiaire [*T*_Fus._ = 451 (1) K]. Après refroidissement du mélange, un fragment monocristallin pris du solide obtenu a été utilisé pour la collecte des données.

## Affinement   

Les données cristallographiques, les conditions de la collecte et de l’affinement sont résumées au Tableau 1 ▸. La localisation des deux cations alcalins a été basée sur leurs densités électroniques différentes ainsi que sur leurs distances par rapport aux atomes d’oxygène. Leurs taux d’occupation n’ont pas dévié de l’unité, excluant ainsi la possibilité d’existence d’un désordre de substitution. Le résidu électronique final, de 0.59 e Å^−3^, se situe à 0.93 Å de Rb1. Malgré un nombre faible de paires de Friedel, mais graĉe à la présence de diffuseurs anomales la configuration absolue comme présentée ici semble être la bonne.

## Supplementary Material

Crystal structure: contains datablock(s) global, I. DOI: 10.1107/S2056989015006532/vn2090sup1.cif

Structure factors: contains datablock(s) I. DOI: 10.1107/S2056989015006532/vn2090Isup2.hkl

CCDC reference: 1004334

Additional supporting information:  crystallographic information; 3D view; checkCIF report

## Figures and Tables

**Table 1:** Dtails exprimentaux

Donnes crystallines	
Formule chimique	NaRb_2_(NO_3_)_3_
*M*_r_	379,96
Systme cristallin, groupe d’espace	Orthorhombique, *P* *m* *c*2_1_
Temprature (K)	293
*a*, *b*, *c* ()	5,327(5), 9,079(4), 9,718(6)
*V* (^3^)	470,0(6)
*Z*	2
Type de rayonnement	Mo *K*
(mm^1^)	10,50
Taille des cristaux (mm)	0,4 0,4 0,3

Collection de donnes	
Diffractomtre	EnrafNonius CAD-4
Correction d’absorption	scan (North *et al.*, 1968 ▸)
*T*_min_, *T*_max_	0,545, 0,995
Nombre de rflexions mesures, indpendantes et observes [*I* > 2(*I*)]	1474, 727, 662
*R*_int_	0,038
(sin /)_max_ (^1^)	0,660

Affinement	
*R*[*F* ^2^ > 2(*F* ^2^)], *wR*(*F* ^2^), *S*	0,024, 0,062, 0,83
Nombre de rflexions	727
Nombre de paramtres	83
Nombre de restraints	1
_max_, _min_ (e ^3^)	0,59, 0,56
Absolute structure	Flack (1983 ▸), 63 paires de Friedel
Paramtre de structure absolue	0.009(16)

Programmes informatiques: *CAD-4 EXPRESS* (Duisenberg, 1992 ▸; Macek Yordanov, 1992 ▸), *XCAD4* (Harms Wocadlo, 1995 ▸), *SHELXS97* et *SHELXL97* (Sheldrick, 2008 ▸), *DIAMOND* (Brandenburg, 2006 ▸), *WinGX* (Farrugia, 2012 ▸) et *publCIF* (Westrip, 2010 ▸).

## supplementary crystallographic information

### Crystal data

**Table:**

NaRb_2_(NO_3_)_3_	*F*(000) = 356
*M**_r_* = 379.96	*D*_x_ = 2.685 Mg m^−^^3^
Orthorhombic, *P**m**c*2_1_	Mo *K*α radiation, λ = 0.71073 Å
Hall symbol: P 2c -2	Cell parameters from 25 reflections
*a* = 5.327 (5) Å	θ = 10–15°
*b* = 9.079 (4) Å	µ = 10.50 mm^−^^1^
*c* = 9.718 (6) Å	*T* = 293 K
*V* = 470.0 (6) Å^3^	Parallelepiped, white
*Z* = 2	0.4 × 0.4 × 0.3 mm

### Data collection

**Table:**

Enraf–Nonius CAD-4 diffractometer	*R*_int_ = 0.038
Graphite monochromator	θ_max_ = 28.0°, θ_min_ = 2.2°
ω/2θ scans	*h* = −7→7
Absorption correction: ψ scan (North *et al.*, 1968)	*k* = −1→11
*T*_min_ = 0.545, *T*_max_ = 0.995	*l* = −1→12
1474 measured reflections	2 standard reflections every 120 reflections
727 independent reflections	intensity decay: 4%
662 reflections with *I* > 2σ(*I*)	

### Refinement

**Table:**

Refinement on *F*^2^	*w* = 1/[σ^2^(*F*_o_^2^) + (0.0532*P*)^2^] where *P* = (*F*_o_^2^ + 2*F*_c_^2^)/3
Least-squares matrix: full	(Δ/σ)_max_ < 0.001
*R*[*F*^2^ > 2σ(*F*^2^)] = 0.024	Δρ_max_ = 0.59 e Å^−^^3^
*wR*(*F*^2^) = 0.062	Δρ_min_ = −0.56 e Å^−^^3^
*S* = 0.83	Extinction correction: (*SHELXL97*; Sheldrick, 2008)
727 reflections	Extinction coefficient: 0.036 (3)
83 parameters	Absolute structure: Flack (1983), 63 paires de Friedel
1 restraint	Absolute structure parameter: −0.009 (16)

### Special details

**Table:**

Geometry. All e.s.d.'s (except the e.s.d. in the dihedral angle between two l.s. planes) are estimated using the full covariance matrix. The cell e.s.d.'s are taken into account individually in the estimation of e.s.d.'s in distances, angles and torsion angles; correlations between e.s.d.'s in cell parameters are only used when they are defined by crystal symmetry. An approximate (isotropic) treatment of cell e.s.d.'s is used for estimating e.s.d.'s involving l.s. planes.

### Fractional atomic coordinates and isotropic or equivalent isotropic displacement parameters (Å^2^)

**Table:**

	*x*	*y*	*z*	*U*_iso_*/*U*_eq_	
Rb1	0.5	0.08278 (8)	0.33461 (7)	0.0293 (2)	
Rb2	0.5	0.37837 (8)	0.66067 (7)	0.0312 (2)	
Na1	0	0.7007 (3)	0.6151 (3)	0.0329 (8)	
N1	1	0.0537 (7)	0.6267 (7)	0.0292 (15)	
O1	1	−0.0580 (6)	0.7033 (7)	0.0382 (14)	
O2	0.7945 (12)	0.1047 (6)	0.5920 (7)	0.0665 (17)	
N2	0	0.4139 (7)	0.4141 (8)	0.0263 (14)	
O3	0	0.4581 (7)	0.5370 (6)	0.0374 (14)	
O4	0.2020 (10)	0.3901 (5)	0.3557 (7)	0.0615 (16)	
N3	0.5	0.2516 (6)	0.9835 (7)	0.0268 (13)	
O5	0.5	0.2981 (6)	1.1048 (6)	0.0391 (14)	
O6	0.7029 (8)	0.2278 (5)	0.9225 (5)	0.0443 (11)	

### Atomic displacement parameters (Å^2^)

**Table:**

	*U*^11^	*U*^22^	*U*^33^	*U*^12^	*U*^13^	*U*^23^
Rb1	0.0286 (4)	0.0330 (4)	0.0263 (4)	0	0	0.0045 (3)
Rb2	0.0313 (4)	0.0257 (3)	0.0366 (4)	0	0	0.0044 (3)
Na1	0.0323 (15)	0.0263 (15)	0.0400 (19)	0	0	−0.0054 (13)
N1	0.037 (3)	0.022 (3)	0.029 (4)	0	0	−0.006 (3)
O1	0.045 (3)	0.024 (2)	0.046 (4)	0	0	0.005 (2)
O2	0.066 (3)	0.067 (3)	0.067 (4)	0.032 (3)	−0.027 (3)	−0.006 (3)
N2	0.027 (3)	0.023 (3)	0.030 (4)	0	0	−0.002 (3)
O3	0.050 (4)	0.033 (3)	0.029 (3)	0	0	−0.006 (2)
O4	0.053 (3)	0.062 (3)	0.069 (4)	0.007 (2)	0.032 (3)	0.000 (3)
N3	0.029 (3)	0.027 (3)	0.025 (3)	0	0	−0.002 (3)
O5	0.057 (4)	0.033 (3)	0.027 (3)	0	0	−0.002 (3)
O6	0.033 (2)	0.059 (3)	0.041 (3)	0.003 (2)	0.007 (2)	−0.007 (2)

### Geometric parameters (Å, º)

**Table:**

Rb1—O2	2.959 (6)	Na1—N3^xii^	2.986 (4)
Rb1—O2^i^	2.959 (6)	Na1—N3^vi^	2.986 (4)
Rb1—O1^ii^	2.962 (4)	Na1—Rb1^viii^	3.938 (3)
Rb1—O1^iii^	2.962 (4)	Na1—Rb1^xiii^	3.938 (3)
Rb1—O5^iv^	2.968 (6)	N1—O2	1.235 (6)
Rb1—O6^ii^	3.138 (5)	N1—O2^xiv^	1.235 (6)
Rb1—O6^v^	3.138 (5)	N1—O1	1.258 (9)
Rb1—O4^i^	3.217 (5)	N1—Rb1^xv^	3.565 (5)
Rb1—O4	3.217 (5)	N1—Rb1^xvi^	3.565 (5)
Rb1—O2^ii^	3.304 (7)	O1—Na1^xvii^	2.352 (6)
Rb1—O2^v^	3.304 (7)	O1—Rb1^xv^	2.962 (4)
Rb1—N3^ii^	3.363 (6)	O1—Rb1^xvi^	2.962 (4)
Rb2—O5^vi^	2.988 (6)	O2—Rb1^xvi^	3.304 (7)
Rb2—O3^vii^	3.010 (4)	N2—O4^xviii^	1.236 (6)
Rb2—O3	3.010 (4)	N2—O4	1.236 (6)
Rb2—O2^i^	3.014 (5)	N2—O3	1.259 (10)
Rb2—O2	3.014 (5)	N2—Rb2^xix^	3.597 (5)
Rb2—O6	3.084 (5)	O3—Rb2^xix^	3.010 (4)
Rb2—O6^i^	3.084 (5)	O4—Na1^xii^	2.702 (7)
Rb2—O4^viii^	3.245 (5)	O4—Rb2^vi^	3.245 (5)
Rb2—O4^ix^	3.245 (5)	N3—O6^i^	1.252 (5)
Rb2—N3	3.341 (7)	N3—O6	1.252 (5)
Rb2—O4^i^	3.364 (7)	N3—O5	1.252 (10)
Rb2—O4	3.364 (7)	N3—Na1^viii^	2.986 (4)
Na1—O3	2.330 (6)	N3—Na1^xiii^	2.986 (4)
Na1—O1^x^	2.352 (6)	N3—Rb1^xvi^	3.363 (6)
Na1—O6^xi^	2.536 (5)	O5—Na1^viii^	2.665 (3)
Na1—O6^vi^	2.536 (5)	O5—Na1^xiii^	2.665 (3)
Na1—O5^xii^	2.665 (3)	O5—Rb1^xx^	2.968 (6)
Na1—O5^vi^	2.665 (3)	O5—Rb2^viii^	2.988 (6)
Na1—O4^ix^	2.702 (7)	O6—Na1^viii^	2.536 (5)
Na1—O4^xiii^	2.702 (7)	O6—Rb1^xvi^	3.138 (5)
			
O2—Rb1—O2^i^	64.0 (3)	O3—Na1—O5^vi^	89.52 (14)
O2—Rb1—O1^ii^	147.78 (18)	O1^x^—Na1—O5^vi^	90.56 (14)
O2^i^—Rb1—O1^ii^	83.83 (19)	O6^xi^—Na1—O5^vi^	126.5 (2)
O2—Rb1—O1^iii^	83.83 (19)	O6^vi^—Na1—O5^vi^	49.27 (16)
O2^i^—Rb1—O1^iii^	147.78 (18)	O5^xii^—Na1—O5^vi^	175.6 (3)
O1^ii^—Rb1—O1^iii^	128.1 (2)	O3—Na1—O4^ix^	89.6 (2)
O2—Rb1—O5^iv^	126.29 (15)	O1^x^—Na1—O4^ix^	88.2 (2)
O2^i^—Rb1—O5^iv^	126.29 (15)	O6^xi^—Na1—O4^ix^	164.85 (17)
O1^ii^—Rb1—O5^iv^	74.08 (13)	O6^vi^—Na1—O4^ix^	117.92 (15)
O1^iii^—Rb1—O5^iv^	74.08 (13)	O5^xii^—Na1—O4^ix^	115.6 (2)
O2—Rb1—O6^ii^	90.74 (14)	O5^vi^—Na1—O4^ix^	68.66 (17)
O2^i^—Rb1—O6^ii^	69.38 (13)	O3—Na1—O4^xiii^	89.6 (2)
O1^ii^—Rb1—O6^ii^	74.89 (14)	O1^x^—Na1—O4^xiii^	88.2 (2)
O1^iii^—Rb1—O6^ii^	111.03 (13)	O6^xi^—Na1—O4^xiii^	117.92 (15)
O5^iv^—Rb1—O6^ii^	142.79 (13)	O6^vi^—Na1—O4^xiii^	164.85 (17)
O2—Rb1—O6^v^	69.38 (13)	O5^xii^—Na1—O4^xiii^	68.66 (17)
O2^i^—Rb1—O6^v^	90.74 (14)	O5^vi^—Na1—O4^xiii^	115.6 (2)
O1^ii^—Rb1—O6^v^	111.03 (13)	O4^ix^—Na1—O4^xiii^	46.9 (2)
O1^iii^—Rb1—O6^v^	74.89 (14)	O3—Na1—N3^xii^	89.85 (16)
O5^iv^—Rb1—O6^v^	142.79 (13)	O1^x^—Na1—N3^xii^	91.21 (15)
O6^ii^—Rb1—O6^v^	40.29 (15)	O6^xi^—Na1—N3^xii^	24.50 (15)
O2—Rb1—O4^i^	68.06 (15)	O6^vi^—Na1—N3^xii^	101.72 (19)
O2^i^—Rb1—O4^i^	98.60 (16)	O5^xii^—Na1—N3^xii^	24.77 (19)
O1^ii^—Rb1—O4^i^	122.48 (14)	O5^vi^—Na1—N3^xii^	151.0 (2)
O1^iii^—Rb1—O4^i^	69.48 (15)	O4^ix^—Na1—N3^xii^	140.35 (18)
O5^iv^—Rb1—O4^i^	58.44 (13)	O4^xiii^—Na1—N3^xii^	93.42 (17)
O6^ii^—Rb1—O4^i^	158.76 (14)	O3—Na1—N3^vi^	89.85 (16)
O6^v^—Rb1—O4^i^	126.31 (13)	O1^x^—Na1—N3^vi^	91.21 (15)
O2—Rb1—O4	98.60 (16)	O6^xi^—Na1—N3^vi^	101.72 (19)
O2^i^—Rb1—O4	68.06 (15)	O6^vi^—Na1—N3^vi^	24.50 (15)
O1^ii^—Rb1—O4	69.48 (15)	O5^xii^—Na1—N3^vi^	151.0 (2)
O1^iii^—Rb1—O4	122.48 (14)	O5^vi^—Na1—N3^vi^	24.77 (19)
O5^iv^—Rb1—O4	58.44 (13)	O4^ix^—Na1—N3^vi^	93.42 (17)
O6^ii^—Rb1—O4	126.31 (13)	O4^xiii^—Na1—N3^vi^	140.35 (18)
O6^v^—Rb1—O4	158.76 (14)	N3^xii^—Na1—N3^vi^	126.2 (3)
O4^i^—Rb1—O4	59.1 (2)	O3—Na1—Rb1^viii^	130.42 (10)
O2—Rb1—O2^ii^	152.81 (8)	O1^x^—Na1—Rb1^viii^	48.53 (8)
O2^i^—Rb1—O2^ii^	112.71 (17)	O6^xi^—Na1—Rb1^viii^	133.97 (16)
O1^ii^—Rb1—O2^ii^	39.33 (14)	O6^vi^—Na1—Rb1^viii^	81.38 (12)
O1^iii^—Rb1—O2^ii^	94.61 (15)	O5^xii^—Na1—Rb1^viii^	133.96 (17)
O5^iv^—Rb1—O2^ii^	78.59 (14)	O5^vi^—Na1—Rb1^viii^	48.89 (14)
O6^ii^—Rb1—O2^ii^	64.39 (14)	O4^ix^—Na1—Rb1^viii^	54.16 (11)
O6^v^—Rb1—O2^ii^	83.97 (13)	O4^xiii^—Na1—Rb1^viii^	87.31 (14)
O4^i^—Rb1—O2^ii^	136.59 (15)	N3^xii^—Na1—Rb1^viii^	139.72 (15)
O4—Rb1—O2^ii^	104.94 (15)	N3^vi^—Na1—Rb1^viii^	63.67 (13)
O2—Rb1—O2^v^	112.71 (17)	O3—Na1—Rb1^xiii^	130.42 (10)
O2^i^—Rb1—O2^v^	152.81 (8)	O1^x^—Na1—Rb1^xiii^	48.53 (8)
O1^ii^—Rb1—O2^v^	94.61 (15)	O6^xi^—Na1—Rb1^xiii^	81.38 (12)
O1^iii^—Rb1—O2^v^	39.33 (14)	O6^vi^—Na1—Rb1^xiii^	133.97 (15)
O5^iv^—Rb1—O2^v^	78.59 (14)	O5^xii^—Na1—Rb1^xiii^	48.89 (14)
O6^ii^—Rb1—O2^v^	83.97 (13)	O5^vi^—Na1—Rb1^xiii^	133.96 (17)
O6^v^—Rb1—O2^v^	64.39 (14)	O4^ix^—Na1—Rb1^xiii^	87.31 (14)
O4^i^—Rb1—O2^v^	104.94 (15)	O4^xiii^—Na1—Rb1^xiii^	54.16 (11)
O4—Rb1—O2^v^	136.59 (15)	N3^xii^—Na1—Rb1^xiii^	63.67 (13)
O2^ii^—Rb1—O2^v^	56.70 (19)	N3^vi^—Na1—Rb1^xiii^	139.72 (15)
O2—Rb1—N3^ii^	72.37 (15)	Rb1^viii^—Na1—Rb1^xiii^	85.12 (9)
O2^i^—Rb1—N3^ii^	72.37 (15)	O2—N1—O2^xiv^	124.8 (8)
O1^ii^—Rb1—N3^ii^	96.71 (12)	O2—N1—O1	117.6 (4)
O1^iii^—Rb1—N3^ii^	96.71 (12)	O2^xiv^—N1—O1	117.6 (4)
O5^iv^—Rb1—N3^ii^	156.66 (15)	O2—N1—Rb1^xv^	161.2 (5)
O6^ii^—Rb1—N3^ii^	21.85 (9)	O2^xiv^—N1—Rb1^xv^	67.9 (4)
O6^v^—Rb1—N3^ii^	21.85 (9)	O1—N1—Rb1^xv^	52.02 (16)
O4^i^—Rb1—N3^ii^	139.09 (12)	O2—N1—Rb1^xvi^	67.9 (4)
O4—Rb1—N3^ii^	139.09 (12)	O2^xiv^—N1—Rb1^xvi^	161.2 (5)
O2^ii^—Rb1—N3^ii^	80.91 (15)	O1—N1—Rb1^xvi^	52.02 (16)
O2^v^—Rb1—N3^ii^	80.91 (15)	Rb1^xv^—N1—Rb1^xvi^	96.68 (18)
O5^vi^—Rb2—O3^vii^	72.01 (12)	N1—O1—Na1^xvii^	122.4 (5)
O5^vi^—Rb2—O3	72.01 (12)	N1—O1—Rb1^xv^	108.42 (16)
O3^vii^—Rb2—O3	124.4 (2)	Na1^xvii^—O1—Rb1^xv^	94.95 (12)
O5^vi^—Rb2—O2^i^	140.40 (15)	N1—O1—Rb1^xvi^	108.42 (16)
O3^vii^—Rb2—O2^i^	124.77 (16)	Na1^xvii^—O1—Rb1^xvi^	94.95 (12)
O3—Rb2—O2^i^	69.46 (17)	Rb1^xv^—O1—Rb1^xvi^	128.1 (2)
O5^vi^—Rb2—O2	140.40 (15)	N1—O2—Rb1	132.5 (5)
O3^vii^—Rb2—O2	69.46 (17)	N1—O2—Rb2	135.2 (5)
O3—Rb2—O2	124.77 (16)	Rb1—O2—Rb2	88.08 (14)
O2^i^—Rb2—O2	62.7 (2)	N1—O2—Rb1^xvi^	91.9 (4)
O5^vi^—Rb2—O6	125.87 (14)	Rb1—O2—Rb1^xvi^	108.5 (2)
O3^vii^—Rb2—O6	97.23 (14)	Rb2—O2—Rb1^xvi^	91.11 (18)
O3—Rb2—O6	138.26 (14)	O4^xviii^—N2—O4	121.1 (9)
O2^i^—Rb2—O6	89.98 (15)	O4^xviii^—N2—O3	119.4 (4)
O2—Rb2—O6	68.58 (15)	O4—N2—O3	119.4 (4)
O5^vi^—Rb2—O6^i^	125.87 (14)	O4^xviii^—N2—Rb2	159.2 (5)
O3^vii^—Rb2—O6^i^	138.26 (13)	O4—N2—Rb2	69.2 (4)
O3—Rb2—O6^i^	97.23 (14)	O3—N2—Rb2	52.92 (18)
O2^i^—Rb2—O6^i^	68.58 (15)	O4^xviii^—N2—Rb2^xix^	69.2 (4)
O2—Rb2—O6^i^	89.98 (15)	O4—N2—Rb2^xix^	159.3 (5)
O6—Rb2—O6^i^	41.04 (16)	O3—N2—Rb2^xix^	52.92 (18)
O5^vi^—Rb2—O4^viii^	57.95 (13)	Rb2—N2—Rb2^xix^	95.54 (18)
O3^vii^—Rb2—O4^viii^	69.19 (16)	N2—O3—Na1	127.6 (5)
O3—Rb2—O4^viii^	120.69 (15)	N2—O3—Rb2	107.6 (2)
O2^i^—Rb2—O4^viii^	156.66 (17)	Na1—O3—Rb2	95.57 (15)
O2—Rb2—O4^viii^	114.13 (14)	N2—O3—Rb2^xix^	107.6 (2)
O6—Rb2—O4^viii^	68.52 (13)	Na1—O3—Rb2^xix^	95.57 (15)
O6^i^—Rb2—O4^viii^	88.67 (15)	Rb2—O3—Rb2^xix^	124.4 (2)
O5^vi^—Rb2—O4^ix^	57.95 (13)	N2—O4—Na1^xii^	96.0 (5)
O3^vii^—Rb2—O4^ix^	120.69 (15)	N2—O4—Rb1	127.6 (4)
O3—Rb2—O4^ix^	69.19 (16)	Na1^xii^—O4—Rb1	82.92 (15)
O2^i^—Rb2—O4^ix^	114.13 (14)	N2—O4—Rb2^vi^	125.5 (4)
O2—Rb2—O4^ix^	156.66 (17)	Na1^xii^—O4—Rb2^vi^	83.53 (17)
O6—Rb2—O4^ix^	88.67 (15)	Rb1—O4—Rb2^vi^	106.46 (15)
O6^i^—Rb2—O4^ix^	68.52 (13)	N2—O4—Rb2	90.7 (4)
O4^viii^—Rb2—O4^ix^	58.6 (2)	Na1^xii^—O4—Rb2	160.12 (19)
O5^vi^—Rb2—N3	120.62 (15)	Rb1—O4—Rb2	78.21 (13)
O3^vii^—Rb2—N3	117.24 (11)	Rb2^vi^—O4—Rb2	107.71 (17)
O3—Rb2—N3	117.24 (11)	O6^i^—N3—O6	119.5 (7)
O2^i^—Rb2—N3	85.64 (15)	O6^i^—N3—O5	120.3 (3)
O2—Rb2—N3	85.64 (15)	O6—N3—O5	120.3 (3)
O6—Rb2—N3	22.00 (9)	O6^i^—N3—Na1^viii^	176.6 (4)
O6^i^—Rb2—N3	22.00 (9)	O6—N3—Na1^viii^	57.2 (3)
O4^viii^—Rb2—N3	71.02 (15)	O5—N3—Na1^viii^	63.11 (13)
O4^ix^—Rb2—N3	71.02 (14)	O6^i^—N3—Na1^xiii^	57.2 (3)
O5^vi^—Rb2—O4^i^	78.96 (13)	O6—N3—Na1^xiii^	176.6 (4)
O3^vii^—Rb2—O4^i^	39.02 (14)	O5—N3—Na1^xiii^	63.11 (13)
O3—Rb2—O4^i^	93.33 (15)	Na1^viii^—N3—Na1^xiii^	126.2 (3)
O2^i^—Rb2—O4^i^	94.40 (14)	O6^i^—N3—Rb2	67.3 (4)
O2—Rb2—O4^i^	65.50 (16)	O6—N3—Rb2	67.3 (4)
O6—Rb2—O4^i^	125.14 (12)	O5—N3—Rb2	140.2 (4)
O6^i^—Rb2—O4^i^	155.01 (12)	Na1^viii^—N3—Rb2	110.63 (14)
O4^viii^—Rb2—O4^i^	105.26 (15)	Na1^xiii^—N3—Rb2	110.63 (14)
O4^ix^—Rb2—O4^i^	136.46 (9)	O6^i^—N3—Rb1^xvi^	68.9 (3)
N3—Rb2—O4^i^	146.94 (11)	O6—N3—Rb1^xvi^	68.9 (3)
O5^vi^—Rb2—O4	78.96 (13)	O5—N3—Rb1^xvi^	135.1 (5)
O3^vii^—Rb2—O4	93.33 (15)	Na1^viii^—N3—Rb1^xvi^	108.37 (13)
O3—Rb2—O4	39.02 (14)	Na1^xiii^—N3—Rb1^xvi^	108.37 (13)
O2^i^—Rb2—O4	65.50 (16)	Rb2—N3—Rb1^xvi^	84.67 (16)
O2—Rb2—O4	94.40 (14)	N3—O5—Na1^viii^	92.12 (15)
O6—Rb2—O4	155.01 (12)	N3—O5—Na1^xiii^	92.12 (15)
O6^i^—Rb2—O4	125.14 (12)	Na1^viii^—O5—Na1^xiii^	175.6 (3)
O4^viii^—Rb2—O4	136.46 (9)	N3—O5—Rb1^xx^	119.1 (4)
O4^ix^—Rb2—O4	105.26 (14)	Na1^viii^—O5—Rb1^xx^	88.53 (15)
N3—Rb2—O4	146.94 (11)	Na1^xiii^—O5—Rb1^xx^	88.53 (15)
O4^i^—Rb2—O4	56.31 (18)	N3—O5—Rb2^viii^	120.2 (5)
O3—Na1—O1^x^	177.7 (3)	Na1^viii^—O5—Rb2^viii^	89.37 (14)
O3—Na1—O6^xi^	90.1 (2)	Na1^xiii^—O5—Rb2^viii^	89.37 (14)
O1^x^—Na1—O6^xi^	91.76 (19)	Rb1^xx^—O5—Rb2^viii^	120.7 (2)
O3—Na1—O6^vi^	90.1 (2)	N3—O6—Na1^viii^	98.3 (4)
O1^x^—Na1—O6^vi^	91.76 (19)	N3—O6—Rb2	90.7 (4)
O6^xi^—Na1—O6^vi^	77.2 (2)	Na1^viii^—O6—Rb2	135.6 (2)
O3—Na1—O5^xii^	89.52 (14)	N3—O6—Rb1^xvi^	89.2 (4)
O1^x^—Na1—O5^xii^	90.56 (14)	Na1^viii^—O6—Rb1^xvi^	130.22 (19)
O6^xi^—Na1—O5^xii^	49.27 (16)	Rb2—O6—Rb1^xvi^	93.04 (12)
O6^vi^—Na1—O5^xii^	126.5 (2)		

Symmetry codes: (i) −*x*+1, *y*, *z*; (ii) −*x*+1, −*y*, *z*−1/2; (iii) −*x*+2, −*y*, *z*−1/2; (iv) *x*, *y*, *z*−1; (v) *x*, −*y*, *z*−1/2; (vi) −*x*+1, −*y*+1, *z*−1/2; (vii) *x*+1, *y*, *z*; (viii) −*x*+1, −*y*+1, *z*+1/2; (ix) *x*, −*y*+1, *z*+1/2; (x) *x*−1, *y*+1, *z*; (xi) *x*−1, −*y*+1, *z*−1/2; (xii) −*x*, −*y*+1, *z*−1/2; (xiii) −*x*, −*y*+1, *z*+1/2; (xiv) −*x*+2, *y*, *z*; (xv) −*x*+2, −*y*, *z*+1/2; (xvi) −*x*+1, −*y*, *z*+1/2; (xvii) *x*+1, *y*−1, *z*; (xviii) −*x*, *y*, *z*; (xix) *x*−1, *y*, *z*; (xx) *x*, *y*, *z*+1.
